# Improvement of Photocatalytic and Photodegradable ZnSe Nanorods by a Vulcanization Strategy

**DOI:** 10.1021/acsomega.4c05603

**Published:** 2024-11-28

**Authors:** Long Chen, Kai Ou, Zhaosen Fan, Lingyu Liu, Fanggong Cai, Yudong Xia, Hongyan Wang

**Affiliations:** † School of Physical Science and Technology, Southwest Jiaotong University, Chengdu 610031, Sichuan, China; ‡ Key Laboratory of Materials and Surface Technology (Ministry of Education), School of Materials Science and Engineering, Xihua University, Chengdu 610039, Sichuan, China

## Abstract

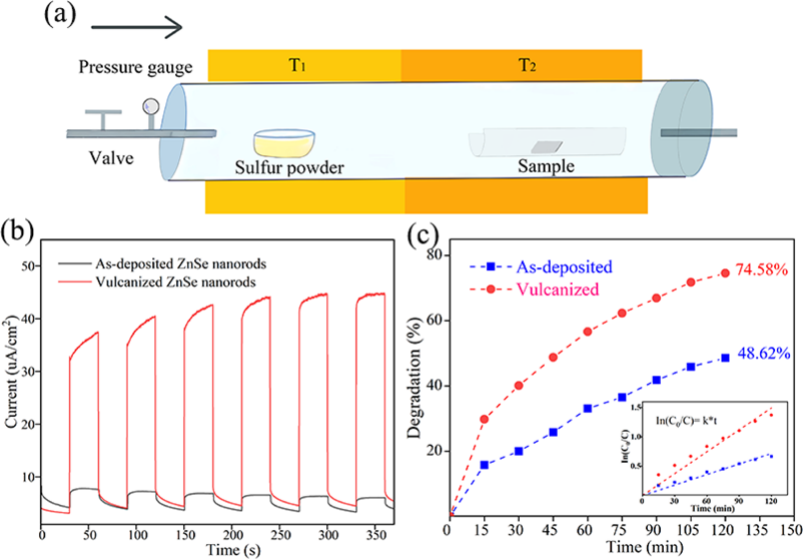

Photocatalysts composed of ZnSe nanorods were prepared by using a glancing angle deposition technique facilitated by electron beam evaporation equipment. To enhance the photocatalytic efficiency of ZnSe, a vulcanization process was introduced. The impact of various parameters, including curing temperature, duration, and nanorod length, on the photocatalytic performance was systematically examined. Comprehensive analysis using X-ray diffraction, scanning electron microscopy, and photocurrent density–potential curves identified optimal vulcanization conditions at 300 °C for 45 min for 170 nm ZnSe nanorods. Under these conditions, the photocurrent reached 44.53 μA/cm^2^, approximately 7-fold greater than that of untreated ZnSe nanorods. Furthermore, the degradation efficiency of Rhodamine B increased by 50%. Detailed analysis of the photocatalytic mechanism revealed that sulfurization not only enhances light absorption but also facilitates the separation of photogenerated carriers through the formation of ZnS.

## 1 Introduction

Over recent decades, the accelerating pace of high-quality industrialization has sharply highlighted the issue of energy shortages and environmental pollution. For environmental issues, gas sensors^1−3^ and organic degradation^4^ have attracted a lot of attention. For the energy problem, the development of new energy sources is very important. Research indicates that semiconductor photocatalysts generating hydrogen, a form of clean energy, may mitigate this challenge.^5,6^ Despite this potential, challenges such as low efficiency, high costs, and instability continue to impede advances in the field of photocatalysis, prompting a series of investigations into materials like ZnO,^7^ TiO_2_,^8^ Fe_2_O_3_,^9^ CdS,^10^ Mxene,^11,12^ and CdSe.^13^ Among these, chalcogenide semiconductor photocatalysts, particularly CdS and CdSe nanocrystals, demonstrate promising catalytic activities and stabilities.^14^ Su et al. synthesized a heterogeneous CdS-Au composite photocatalyst via a facile water bath heating and reflux condensation method for organic pollutants degradation.^15^ However, the inherent toxicity and carcinogenic potential of Cd significantly limit their practical applications.^16^ Consequently, the development of nontoxic photocatalytic materials has emerged as a critical area of research.

ZnSe, classified under the group II–VI compounds, exhibits a direct band gap of ∼2.7 eV, which allows it to effectively absorb both UV and visible light irradiation.^17^ Its low cost, minimal toxicity, and natural abundance significantly enhance its applicability in visible light catalysis. To further improve the photocatalytic performance of ZnSe, various strategies have been adopted. These include different morphologies such as nanoparticles, nanowires, nanobelts/nanoribbons, nanosheets, nanotubes, and core/shell nanostructures. Notably, cubic zinc blende ZnSe, which shows a degradation rate 1.8 times that of hexagonal wurtzite ZnSe, was prepared using NaBH_4_ in a similar two-step process;^18^ Ning et al. enhanced active sites by controlling the incorporation of molecular clusters, thereby influencing the size and shape of ZnSe nanowires.^19^ Beena et al. utilized a straightforward coprecipitation method to produce ZnSe nanoparticles.^20^ Furthermore, heterojunctions involving ZnSe have been investigated. Ehsan et al. reported the synthesis of ZnO/ZnSe heterostructures through a one-pot hydrothermal method;^21^ Feng et al. developed a Z-type CdSe/ZnSe heterojunction using a simple mechanical mixing strategy;^22^ Vempuluru et al. proposed a facile chemical process for the synthesis of ZnS/ZnSe composites, providing theoretical and experimental insights into their enhanced sunlight-driven photocatalytic H_2_ production.^23^ Wei et al. introduced a novel ZnSe/TiO_2_ nanorod heterojunction photocatalyst utilizing the physical method of glancing angle deposition technology (GLAD).^24^ These heterojunction materials exhibited higher photocatalytic efficiency than pure ZnSe, attributed to the enhanced light absorption and carrier separation offered by the heterojunction structure.^25−27^ The vulcanization method, known for its simplicity and minimal environmental impact, allows for precise control of various parameters, including temperature, time, and air flow. Despite its advantages, the literature on using ZnSe vulcanization to enhance photocatalytic properties is still sparse.

In this study, the photocatalytic performance of ZnSe was significantly enhanced through the vulcanization of ZnSe nanorods. These nanorods were prepared by using electron beam evaporation technology. By optimizing the vulcanization process, the photocatalytic activity of the vulcanized ZnSe nanorods was substantially improved, achieving a photocurrent density of 44.53 μA/cm^2^, which is approximately 7-fold higher than that of unmodified ZnSe nanorods. Additionally, the vulcanized ZnSe nanorods demonstrated an enhanced photocatalytic degradation of Rhodamine B (RhB) under 365 nm UV light exposure. The mechanisms underlying the photocatalysis and degradation processes were extensively analyzed. Overall, the vulcanization method presents a novel approach for enhancing the photocatalytic efficiency of group II–VI compounds.

## 2 Experimental Section

### 2.1 Preparation of ZnSe Nanorods

In this experiment, ZnSe nanorods were prepared by using electron beam evaporation, leveraging GLAD technology. ZnSe particles with a purity of 99.99% served as the evaporation materials. Various substrates, including silicon wafers, fluorine-doped tin oxide (FTO) glass, and quartz chips, were mounted on the substrate rack. The distance between the substrate and target source was about 25 cm. For the photocatalytic and photodegraded samples, the effective area on the FTO substrates was 5 mm × 5 mm and 20 mm × 20 mm, respectively. During the deposition process, the chamber pressure was meticulously maintained at 9 × 10^–4^ Pa. The three critical parameters for synthesizing nanorods via GLAD (substrate angle, rotation speed, and deposition rate) were optimized to 85°, 1 rpm, and 2 Å/s, respectively. The substrate was kept unheated. Furthermore, nanorods of varying thicknesses (90, 130, 170, and 200 nm) were fabricated by precisely controlling the deposition duration. In addition, 150 nm ZnS films were prepared on a quartz substrate by GLAD to analyze the band structure. The substrate angle, rotation speed, and deposition rate were optimized to 0°, 1 rpm, and 2 Å/s, respectively.

### 2.2 Vulcanization of ZnSe Nanorods

**Figure 1:**
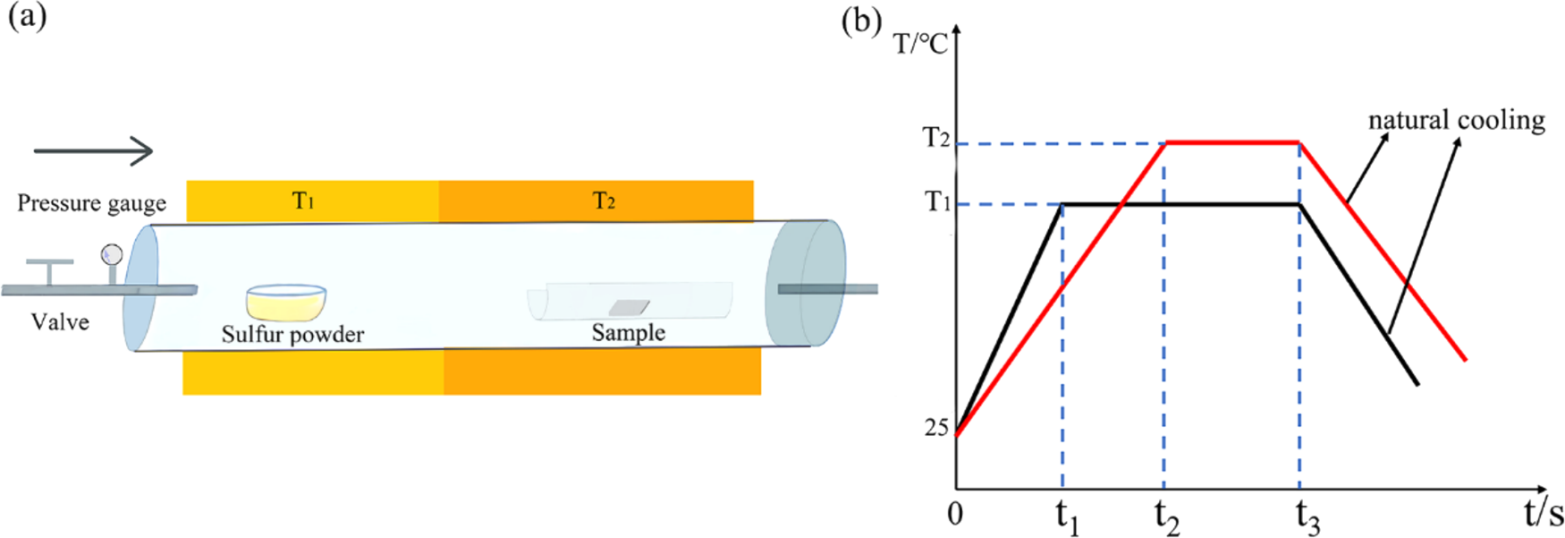
(a) Schematic diagram of the vulcanization and (b) temperature control curves.

The catalyst samples were obtained by sulfurizing ZnSe nanorods in a dual-zone tube furnace, as depicted in Figure 1a. 0.03 g of sulfur powder was placed in the quartz boat in the left warm zone. The ZnSe samples were positioned horizontally in the right temperature zone. The distance was approximately 15 cm apart. During the vulcanization process, argon gas was used as the carrier, with a flow rate of 50 sccm. The temperature control profiles for the two zones are illustrated in Figure 1b. The influence of vulcanization conditions on the heterojunction properties was systematically examined by varying the vulcanization temperature (*T*_2_) and duration (*t*_3_ – *t*_2_). The temperature *T*_1_ in the sulfur zone was held constant at 250 °C, while the temperature *T*_2_ for the sample varied from 250 to 350 °C. The initial duration, *t*_1_, was set at 15 min, and the range for *t*_2_–*t*_3_ varied from 15 to 60 min. Following the treatment, the samples were allowed to cool naturally to room temperature, facilitated by a continuous 50 sccm flow of Ar. The specific conditions applied to different sample series are detailed in Table 1.

**Table 1:** Specific Treatment Conditions for Different Samples

samples	length of the nanorods (nm)	vulcanization temperature (°C)	vulcanization time (min)
S0	170		
SI (A, B, C, D, E)	170	250, 275, 300, 325, 350	45
SII (A, B, C, D)	170	300	15, 30, 45, 60
SIII (A, B, C, D)	90, 130, 170, 200	300	45

### 2.3 Characterization and Testing

X-ray diffraction (XRD) utilizing Cu Kα radiation (λ = 0.154 nm) was employed to analyze the structural characteristics of samples on a silicon wafer substrate. The XRD patterns were scanned from 10 to 60° at intervals of 0.02°. The morphologies of the samples were examined by using field emission scanning electron microscopy (SEM). The contents of different elements in the films were measured by energy-dispersive X-ray spectroscopy (EDS). Absorption spectra, ranging from 200 to 800 nm, were analyzed using a UV spectrophotometer. Compositional and surface properties of the nanostructured materials were characterized by X-ray photoelectron spectroscopy (XPS). Photocatalytic properties were assessed using a conventional three-electrode system under simulated AM 1.5G irradiation (xenon lamp, 100 mW/cm^2^), employing 10 mm × 10 mm Pt foil as the counter electrode, Ag/AgCl as the reference electrode, and the sample as the working electrode. The light intensity at sample electrodes was 100 mW/cm^2^. The operating voltage range was set from −0.6 to 0.5 V (vs Ag/Cl). Photocatalytic performance was evaluated by alternating light exposures every 5 s at a scan rate of 0.01 V/s. The reaction kinetics and stability were assessed at 30 s intervals of light switching, starting from an initial potential of 0.23 V (vs Ag/Cl). The Mott–Schottky (MS) curve of the sample was determined under illumination at an amplitude of 5 mV and a frequency of 1000 Hz. Furthermore, the concentration of RhB in solution was quantified by using a UV–vis spectrophotometer at 454 nm by measuring its absorbance and comparing it to that of the initial solution (5 mg/mL). The degradation process was carried out at 365 nm UV light intensity of 20 mW/cm^2^. The degradation efficiency was calculated using the following formula:^28^
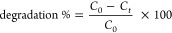
1where *C*_0_ and *C_t_* are the initial and actual concentrations at the given time of RhB, respectively. All of the tests were conducted at room temperature.

## 3 Results and Discussion

### 3.1 Structure and Optical Properties

**Figure 2:**
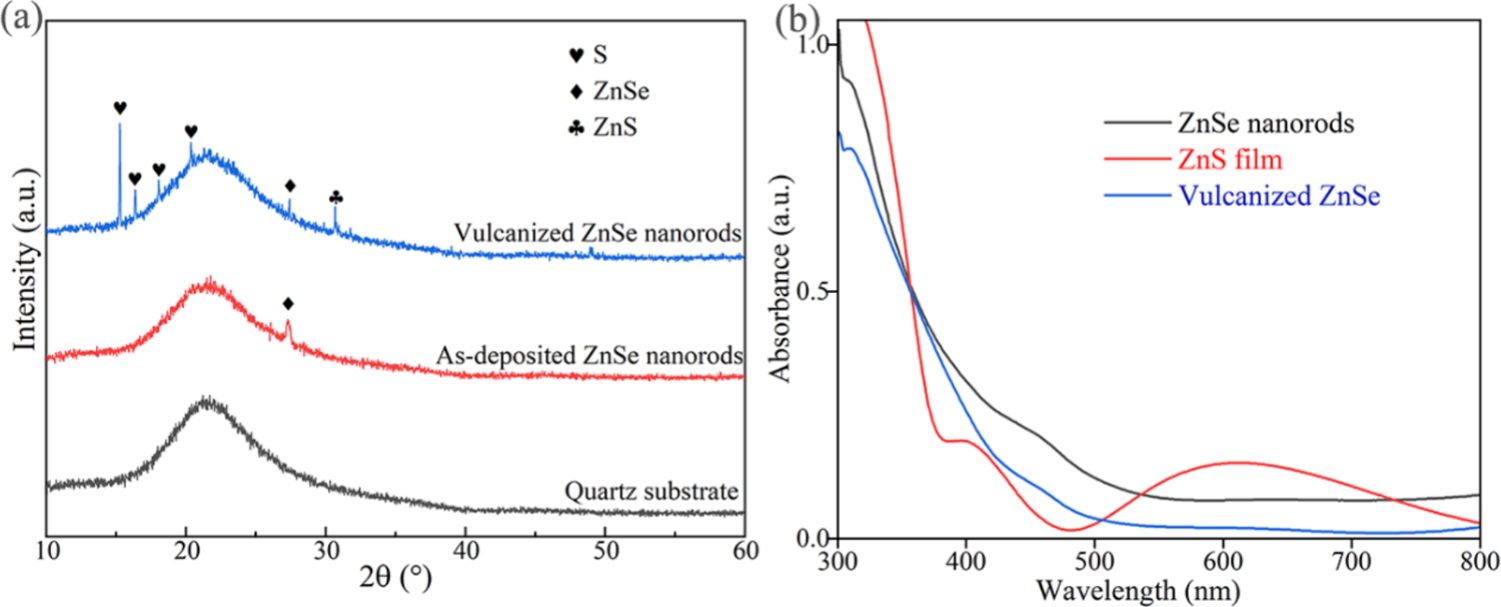
(a) XRD patterns and (b) absorption spectra of ZnS film and as-deposited and vulcanized ZnSe nanorods at 300 °C for 45 min (S0 and SI-C).

The crystal structures of quartz substrates and both as-deposited and vulcanized ZnSe nanorods were characterized by XRD, as shown in Figure 2a. The as-deposited ZnSe nanorods exhibited a diffraction peak at 27.4°, corresponding to the (002) plane of ZnSe (JCPDS: 88-0008). For the vulcanized samples treated at 300 °C, a new peak emerged at 30.7°, attributed to the (101) plane of ZnS (JCPDS: 77-1534), alongside the existing ZnSe peak. Additionally, peaks indicative of elemental sulfur were observed, suggesting the presence of residual sulfur and the formation of a small amount of ZnS in the vulcanized samples. The optical properties of pure ZnSe films and both pure and vulcanized ZnSe nanorods were analyzed via UV–vis absorption spectroscopy and are presented in Figure 2b. The absorption spectra of the as-deposited and vulcanized ZnSe nanorods displayed similar features, with differences noted in the position of the absorption edge and overall absorbance. The increased absorbance in the vulcanized nanorods may enhance their activity under visible-light-driven PHE.

**Figure 3:**
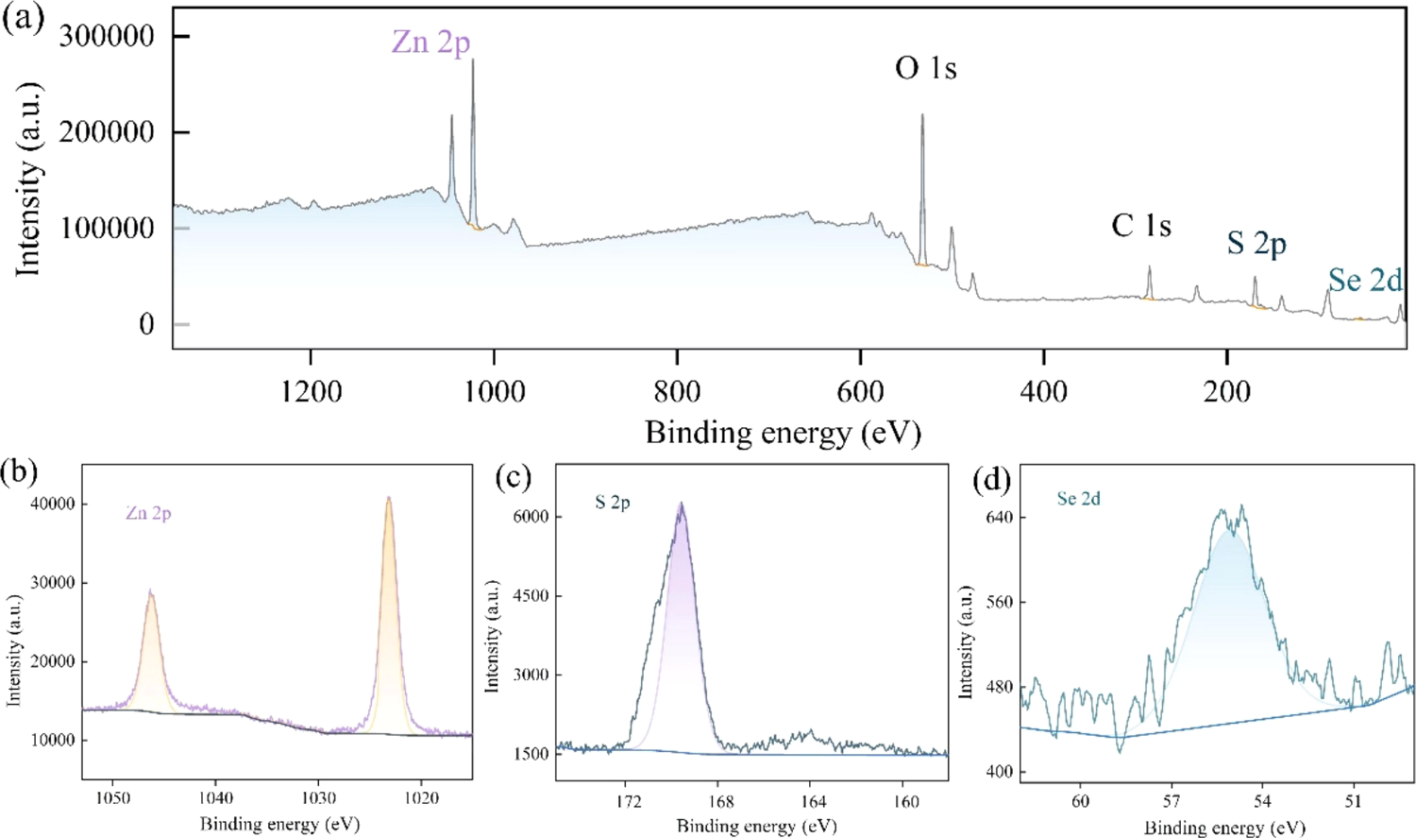
(a) XPS full-scan spectrum of vulcanized ZnSe nanorods (SI-C) and the high-resolution XPS spectra of (b) Zn 2p, (c) S 2p, and (d) Se 2d.

The chemical valence states of elements in the samples were analyzed using XPS. As depicted in Figure 3a, the comprehensive XPS spectrum confirmed the presence of Zn, Se, S, C, and O peaks. The binding energies were calibrated using the C 1s peak at 284.8 eV from adsorbed carbon as a reference. The peak corresponding to oxygen was observed at 531.0 eV, indicative of chemisorbed oxygen. Figure 3b–d displays the high-resolution XPS spectra for Zn 2p, S 2p, and Se 3d, respectively. The Zn 2p peak at 1021.5 eV is characteristic of Zn^2+^ as found in ZnSe or ZnS. The S 2p and Se 3d peaks were located at 162.09 and 45.07 eV, respectively. These findings align with previous reports on nanostructured ZnSe/ZnS films^29^ and quantum dots,^30^ corroborating the presence of ZnSe and ZnS. Moreover, the formation of the ZnSe/ZnS heterojunction not only enhances light absorption but also significantly improves photocatalytic performance.^31^

### 3.2 Photocatalytic Properties and Mechanism of Vulcanized Samples

**Figure 4:**
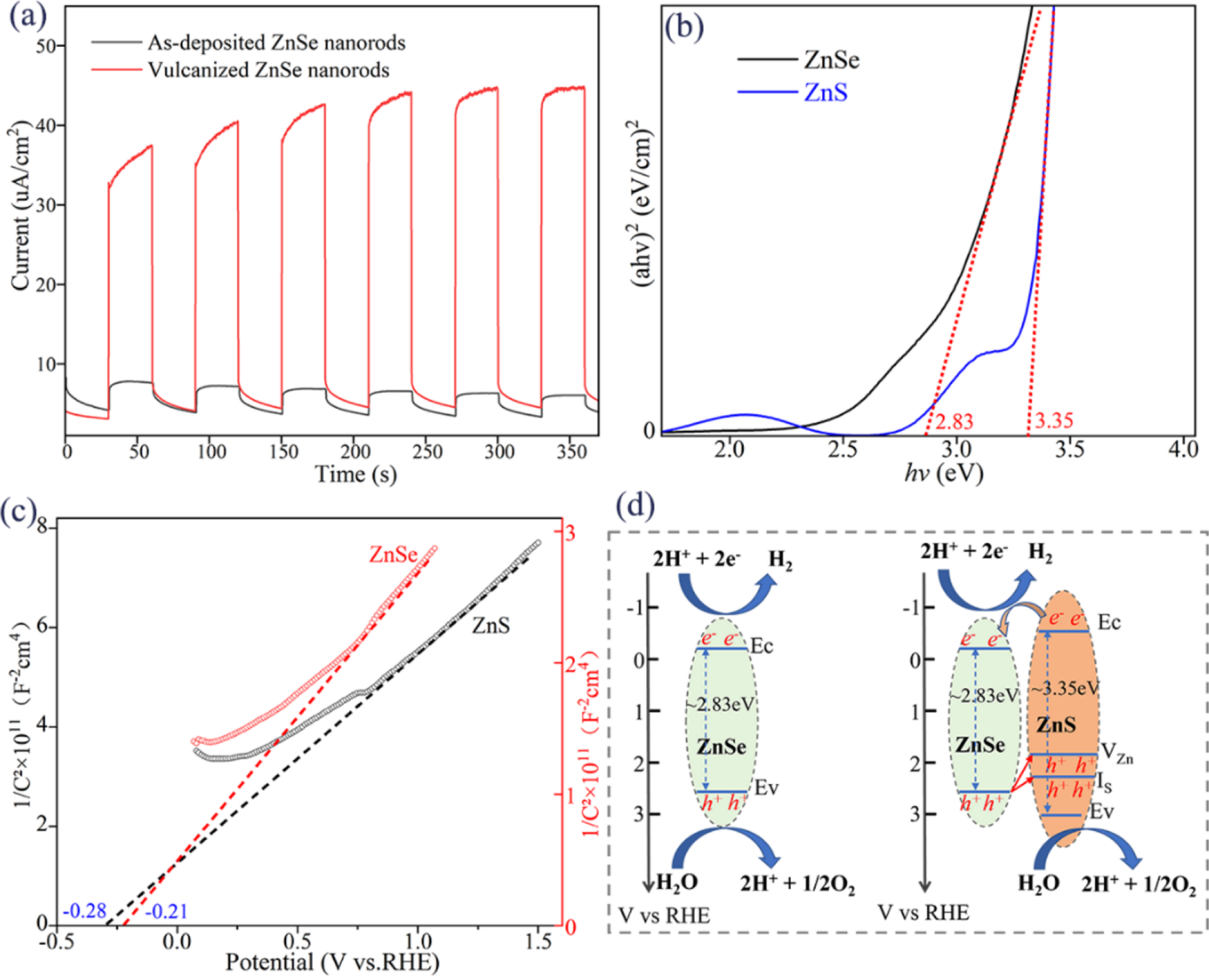
(a) Comparison of photocurrent, (b) optical band gap, (c) flat-band potentials, and (d) photocatalytic mechanism of samples before and after vulcanization at 300 °C for 45 min.

Figure 4a presents the photocurrent density of both as-deposited and vulcanized ZnSe nanorods (SI-C) under a curing condition of 350 °C for 45 min. The maximum photocurrent values reached approximately 44.53 μA/cm^2^ (1.23 V vs RHE) for both types of nanorods, with the photocurrent increasing about 7-fold upon vulcanization. This enhancement suggests that sulfidation of ZnSe is beneficial for improving its photocatalytic performance. Combined with the results of XRD and XPS above, it is necessary to analyze the reason for the performance improvement from the band structure of the heterojunction. Both optical band gap and Nyquist plots of electrochemical impedance spectroscopy were tested. Figure 4b shows the optical band gap of ZnSe and ZnS films, calculated using the following equation:^32^
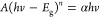
2where *hν*, *E*_g_, and α are photon energy, band gap energy, and absorption coefficient, respectively, and *A* is a proportional constant. As for direct band gap semiconductor *n* = 1/2, the obtained optical band gap energies of ZnSe and ZnS are about 2.83 and 3.35 eV, respectively. Mott–Schottky analysis was used to estimate the charge carrier density and its transfer at the semiconductor–electrolyte interface. The values flat-band potential (*V*_fb_) and carrier concentration (*N*_d_) are measured through the following equation:^12^
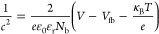
3Here, *N*_d_, *C*, ε_0_, ε_r_, and *k*_B_ represent the charge carrier density, space-charge capacitance of the semiconductor materials, vacuum dielectric constant, relative dielectric constant of samples, and Boltzmann constant, respectively. As shown in Figure 4c, the flat-band potential can be estimated from the intercept of the plot. It can be seen that the flat-band potentials of ZnSe and ZnS are −0.21 and −0.28 V (vs RHE), respectively. The underlying photocatalytic mechanism is depicted in Figure 4d. For unvulcanized ZnSe nanorods, photoexcitation results in the generation of light-induced carriers, where electrons are excited from the valence band to the conduction band, concurrently creating holes in the valence band. The H^+^ ions in the electrolyte capture electrons to form H_2_, while water molecules react with holes (h^+^) to produce H^+^ ions and O_2_, as demonstrated in formulas 4 and 5.^16^ Further analyses via XRD and XPS confirmed the presence of ZnS in the vulcanized samples. The photocatalytic mechanism of vulcanized ZnSe nanorods, illustrated in Figure 4d (right), highlights that ZnSe, known for its narrower band gap compared to ZnS, forms a Type-I heterojunction with ZnS due to ZnSe’s lower conduction band and higher valence band.^33^ Theoretically, electrons can easily transfer from the conduction band of ZnS to that of ZnSe, while holes transfer less readily from the valence band of ZnSe to that of ZnS, which could hinder carrier separation. However, the presence of intrinsic defects in ZnS, such as sulfur vacancies (V_S_), zinc vacancies (V_Zn_), and interstitial sulfur (I_S_),^34^ facilitates this transfer. The energy levels of V_Zn_ and I_S_ are lower than the conduction band of ZnSe,^35^ enabling more effective hole transfer. Consequently, the introduction of ZnS into the structure leads to a more efficient separation of photogenerated carriers, enhancing the photocatalytic performance of the vulcanized ZnSe samples.
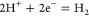
4
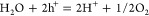
5

### 3.3 Effect of Sulfurization Conditions on Photocatalytic Performance

**Figure 5:**
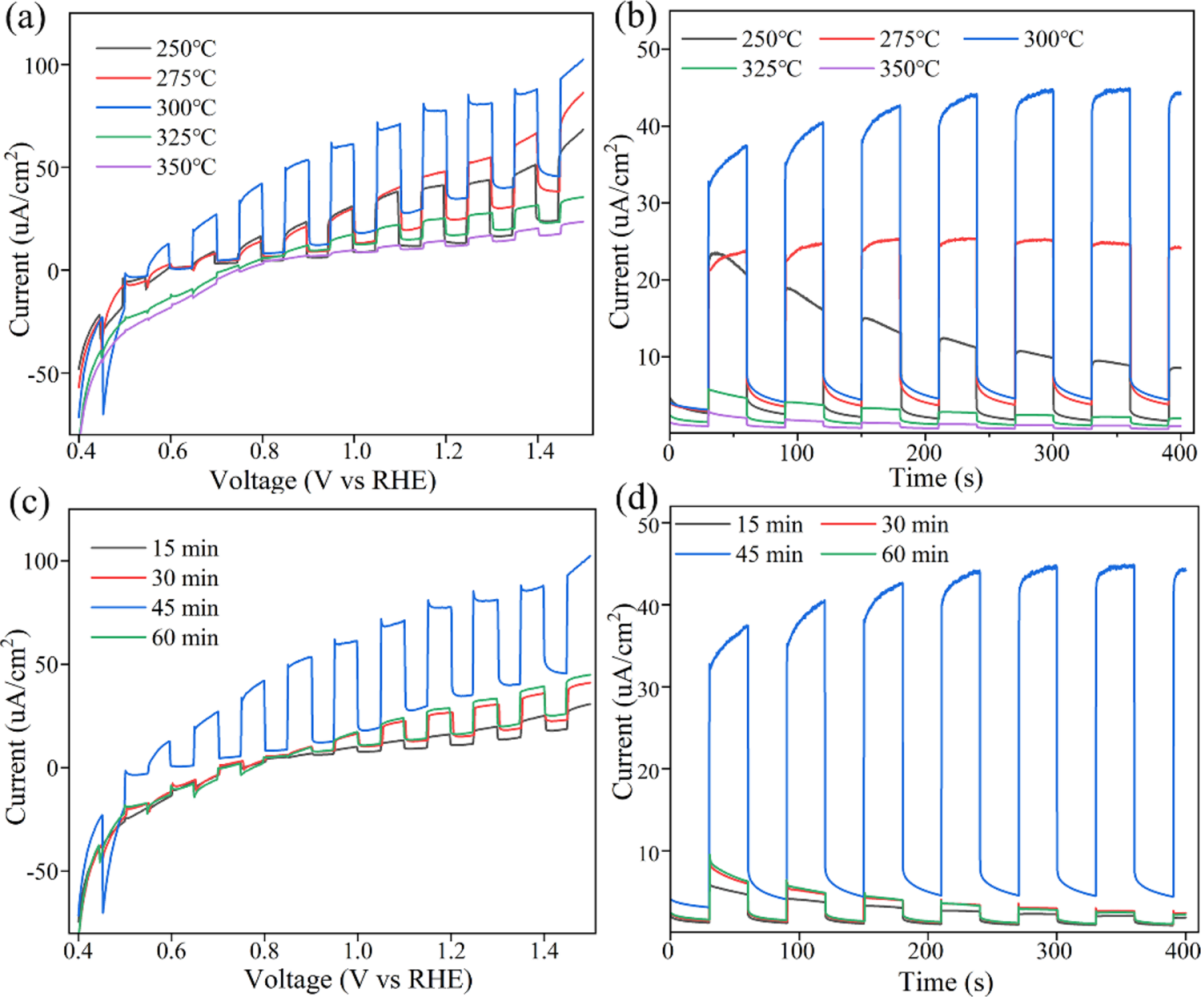
Photocurrent density of samples with different annealing temperatures (sample SI) and times (sample SII): (a, c) different voltages; (b, d) cycle of switching light every 30 s at 1.23 V (vs RHE).

The study explored the influence of vulcanization conditions on photocatalytic performance, focusing on temperature (SI) and duration (SII). Figure 5a,b depicts the photocurrent tests and stability of the photocurrent density for ZnSe samples vulcanized at temperatures ranging from 250 to 350 °C for 45 min. The photocatalytic efficiency of ZnSe nanorods initially increases and subsequently decreases with rising temperatures. The peak photocurrent is observed at 300 °C, registering approximately 44.53 μA/cm^2^ (1.23 V vs RHE). The sample vulcanized at 300 °C not only demonstrates a significant photocurrent but also maintains stability with negligible attenuation. Figure 5c,d presents the photocurrent curves for varying vulcanization durations at 300 °C, with 45 min identified as the optimal time. It is recognized that annealing temperature primarily impacts crystal quality.^36^ Generally, higher annealing temperatures promote better crystallization, enhancing the photocatalytic efficiency, although excessive temperatures may compromise crystalline integrity.

**Figure 6:**
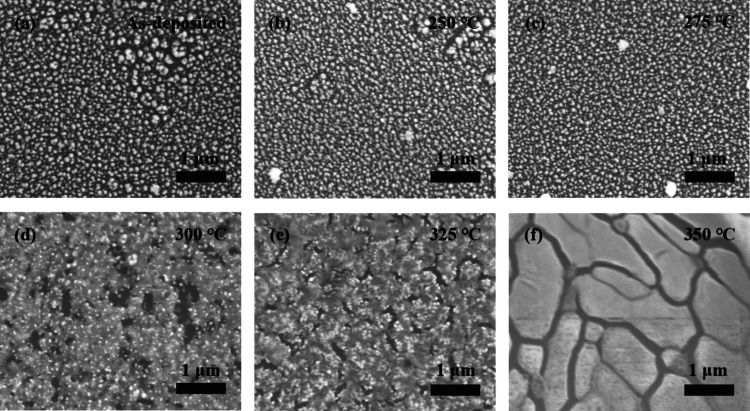
Surface morphologies of as-deposited and vulcanized ZnSe nanorods with various temperatures from 250 to 350 °C (sample SI).

The vulcanization temperature significantly influences the morphology and structure of the samples, as depicted in Figure 6, which presents SEM images of the samples vulcanized for 45 min at various temperatures. Lower temperatures clearly enhance the definition of the nanorods’ structure. As the temperature increases, the vulcanization of the nanorods intensifies, leading to sulfur accumulation on the ZnSe nanorods. Excessive sulfur deposition disrupts the nanorod structure, reducing the number of active sites and diminishing the photocatalytic performance, as illustrated in Figure 6f. Similarly, the curing time impacts sulfur deposition, thereby affecting the active site availability. The elemental content of the samples was also monitored by EDS tests. For samples without vulcanization, the atomic ratio of Zn to Se was 1:0.9, proving the existence of a Se vacancy. Therefore, defects can be remedied by vulcanization. For the vulcanized samples, large differences exist in atomic percentage, mainly S and Zn, but there is very little Se content. This is because EDS can capture only the element proportions of a portion on the surface. From XRD, some crystallization peaks of S indicate that S is still attached to the surface of the sample. As a result, the atomic percentage of the vulcanized sample cannot be accurately obtained. Based on these observations, a vulcanization temperature of 300 °C for 45 min is identified as the optimal condition for our experiments.

It must be noted that the potential limitations or disadvantages of the prepared composites are also determined by the crucial control of vulcanization parameters, including the temperature control of the two temperature zones, the size of the gas flow, the vulcanization time, and the amount of sulfur powder. From the SEM diagram in Figure 6, excessive vulcanization will cause the surface to be covered by a large amount of sulfur, destroy the structure of the nanorods, and lead to a serious decline in catalytic performance. In addition, the morphology of the nanorods also affects the vulcanization effect. Because the nanorods affect the contact area of the sulfur vapor, the ZnSe nanorods and vulcanization conditions need to match each other.

### 3.4 Effect of Film Thickness on Photocatalytic Performance

**Figure 7:**
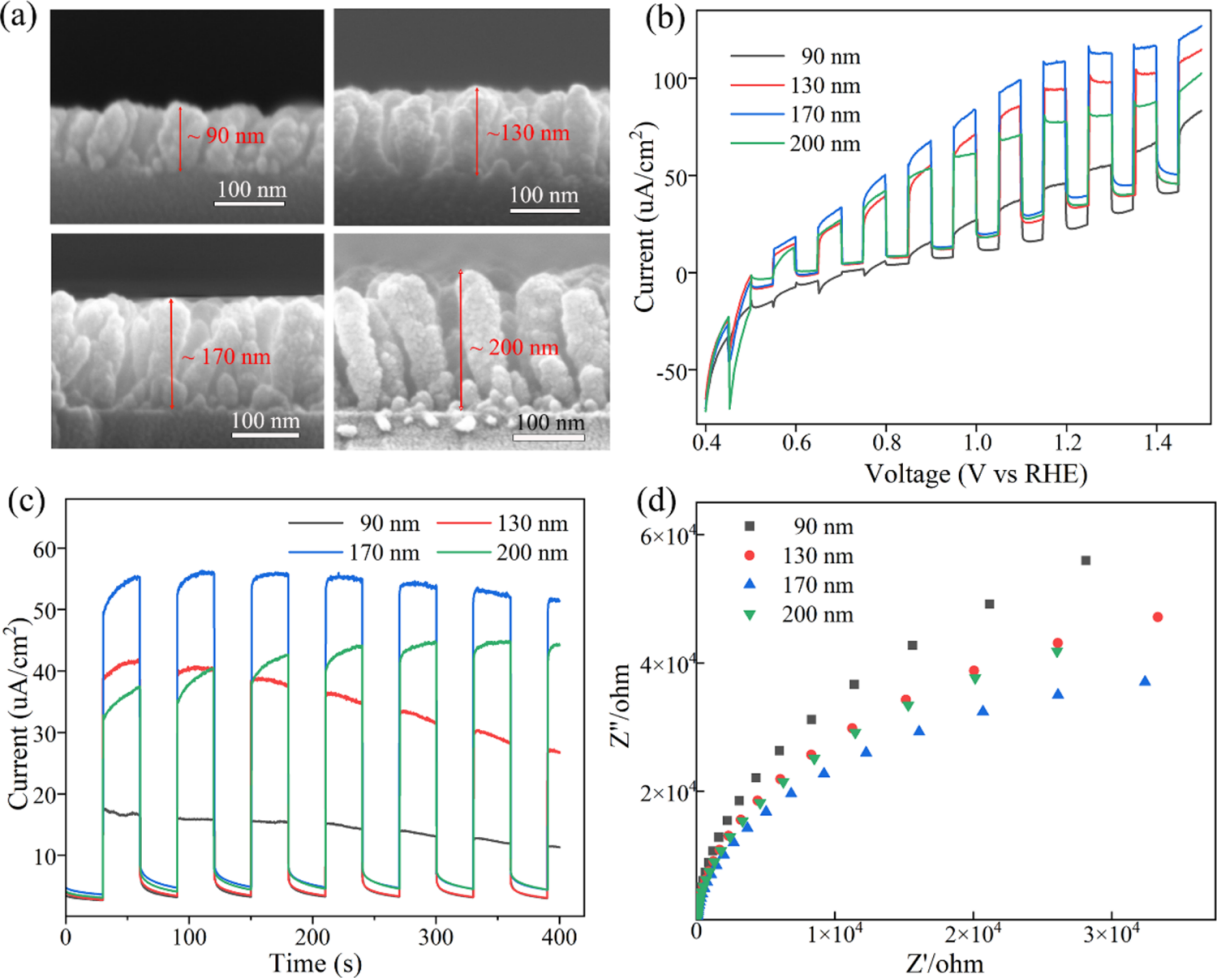
(a) Cross-sectional images, (b, c) photocatalytic performance curves, and (d) electrochemical impedance spectroscopy Nyquist plots of the vulcanized samples (SIII) with different thicknesses.

To explore the influence of the ZnSe nanorod length on photocatalytic properties, ZnSe nanorods of various thicknesses were prepared. These samples underwent vulcanization at 300 °C for 45 min. Figure 7a illustrates the cross sections of samples with thicknesses of approximately 90, 130, 170, and 200 nm. The photocatalytic performance curves are presented in Figure 7b,c. The photocatalytic efficacy generally first increases and then decreases as the thickness of the ZnSe nanorods increases. Typically, extending the length of the nanorods enhances the photocatalytic activity by generating more active sites. However, the performance of the 200 nm sample is lower than that of the 170 nm sample, likely due to insufficient vulcanization. Longer nanorods necessitate extended vulcanization periods at the appropriate temperatures. Optimal vulcanization conditions (300 °C for 45 min) were achieved using 170 nm ZnSe nanorods, as depicted in Figure 7d. Charge transfer capabilities were assessed via electrochemical impedance spectroscopy, where the semicircle indicates the charge transport process with the arc radius correlating to charge transfer resistance.^24^ The results display that the impedance arcs of the sample thickness from 90 to 200 nm are different. According to the EIS data, the 170 nm vulcanized ZnSe nanorods exhibited the lowest Rp value, enabling higher current operation at the same voltage due to the minimal overall charge transfer resistance.

### 3.5 Photodegradation of RhB Solution

**Figure 8:**
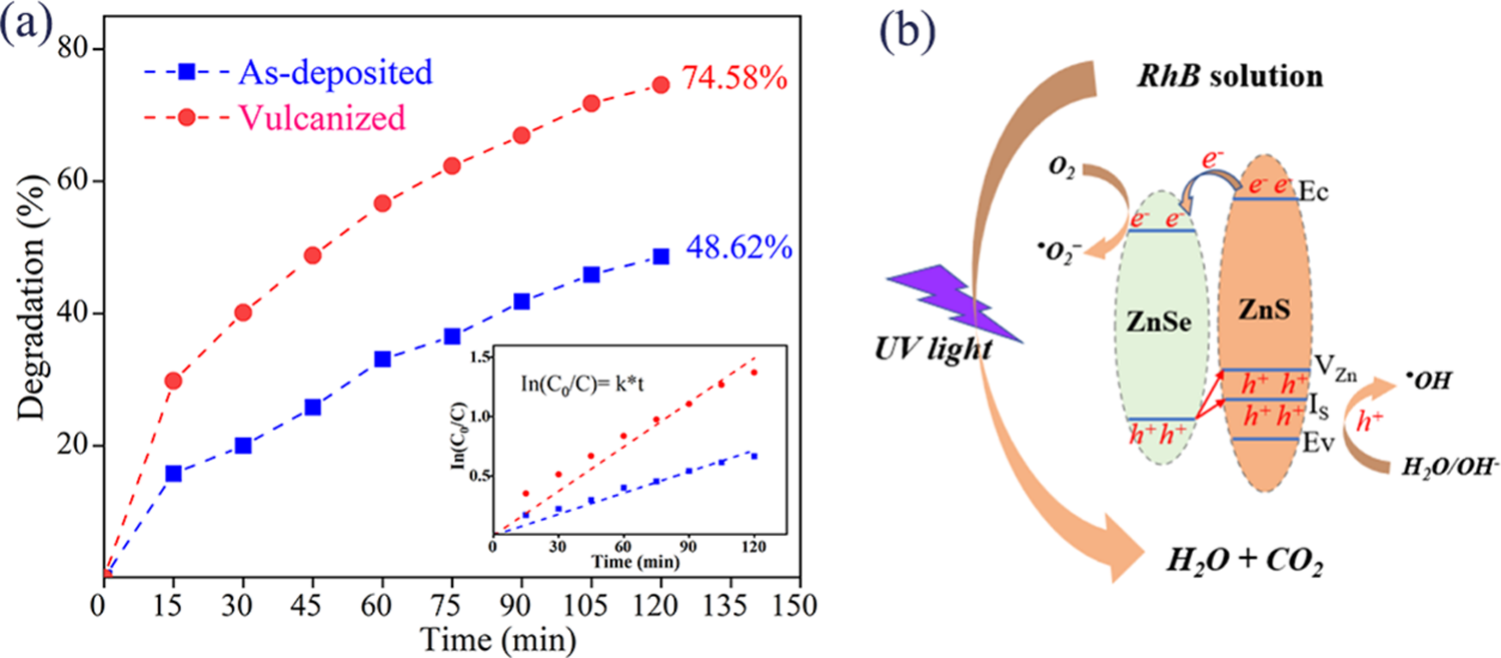
(a) Photocatalytic degradation curves of RhB using as-deposited (sample S0) and vulcanized ZnSe nanorods (sample SII-C) under UV light irradiation; the inset is first-order kinetics plot of the degradation and (b) photocatalysis degradation mechanism of vulcanized samples.

The photocatalytic activities of as-deposited and vulcanized ZnSe nanorods (sample SII-C) were evaluated through the degradation of RhB, a common textile industry pollutant. Degradation efficiency vs reaction time curves for both sample types are depicted in Figure 8a. Postvulcanization, the degradation efficiency reached 74.58%, compared to only 48.62% after 2 h for nonvulcanized ZnSe nanorods, indicating a 1.54-fold increase in photocatalytic activity. The degradation kinetics of RhB under UV light were analyzed by plotting the natural logarithm of the initial concentration over the concentration at time *t* (ln(*C*_0_/*C_t_*)) against irradiation time,^37^ as illustrated in Figure 8a. The linear relationship of ln(*C*_0_/*C_t_*) to the catalytic degradation mechanism of RhB by vulcanized ZnSe nanorods is elucidated in Figure 8b. The vulcanized nanorods exhibit dual functions of absorption and photocatalytic degradation to eliminate RhB from aqueous solutions. Typically, in the absence of light, RhB removal primarily relies on the adsorption capabilities of the material. Under UV irradiation, however, RhB removal necessitates both adsorption and direct photocatalytic degradation on the surface of the contaminant material. The efficiency of photocatalytic degradation largely depends on the material’s photocatalytic properties. Just as analyzed in Figure 4d, vulcanized ZnSe nanorods generate separation electrons and holes under UV irradiation, where electrons are in the conduction band of the ZnSe, and the holes converge in the ZnS. Electrons accumulating effectively reduce adsorbed O_2_ on the surface of the heterojunction to O_2_^–^. Photogenerated h^+^ reacts with H_2_O to form ^•^OH radicals, which then react with RhB to produce CO_2_ and H_2_O, thereby reducing RhB concentration, like other reports.^38,39^ Thus, the photocatalytic degradation of RhB can be described as follows:
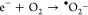
6
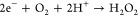
7
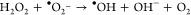
8
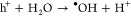
9
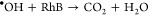
10However, from absorption spectra at 365 nm in Figure 2b, it is found that the absorption intensity of ZnSe nanorods before and after vulcanization is similar. Based on the above analysis, the enhanced degradation efficiency of vulcanized ZnSe nanorods, as discussed, can be ascribed to the effective separation of photogenerated carriers.

## 4 Conclusions

In conclusion, this study introduced a vulcanization approach to enhance the photocatalytic performance of ZnSe nanorods prepared via GLAD technology. The influence of the curing time, temperature, and nanorod length on the catalyst properties was examined comprehensively. The optimal curing conditions were determined to be 300 °C for 45 min. The maximum photocurrent achieved was approximately 44.53 μA/cm^2^, representing a 7-fold increase. The degradation efficiency of RhB improved by 50% compared to that of nonvulcanized ZnSe nanorods. Additionally, the mechanisms underlying photocatalysis and photodegradation were elucidated, with the primary improvements attributed to the role of heterojunctions, leading to carrier separation.

## Data Availability

The data that support the findings of this study are available from the corresponding author upon reasonable request.
